# Identification and characterization of retinoblastoma gene mutations disturbing apoptosis in human breast cancers

**DOI:** 10.1186/1476-4598-9-173

**Published:** 2010-07-01

**Authors:** Elisabet Ognedal Berge, Stian Knappskog, Stephanie Geisler, Vidar Staalesen, Marec Pacal, Anne-Lise Børresen-Dale, Pål Puntervoll, Johan Richard Lillehaug, Per Eystein Lønning

**Affiliations:** 1 Section of Oncology, Institute of Medicine, University of Bergen, Norway; 2 Department of Molecular Biology, University of Bergen, Norway; 3 Department of Oncology, Haukeland University Hospital, Bergen, Norway; 4 Toronto Western Research Institute, University Health Network; 5 Department of Laboratory Medicine and Pathobiology, University of Toronto, Ontario, Canada; 6 Department of Genetics, Institute for Cancer Research, Oslo University Hospital Radiumhospitalet, Oslo, Norway; 7 Institute of Clinical Medicine, Faculty of Medicine, University of Oslo, Norway; 8 Computational Biology Unit, Bergen Center for Computational Science, University of Bergen, Norway; 9 Department of Medicine, Section for Oncology, Asker and Bærum Hospital, 1309 Rud, Norway

## Abstract

**Background:**

The tumor suppressor pRb plays a key role regulating cell cycle arrest, and disturbances in the *RB1 *gene have been reported in different cancer forms. However, the literature reports contradictory findings with respect to a pro - versus anti - apoptotic role of pRb, and the consequence of alterations in *RB1 *to chemotherapy sensitivity remains unclear. This study is part of a project investigating alterations in pivotal genes as predictive factors to chemotherapy sensitivity in breast cancer.

**Results:**

Analyzing 73 locally advanced (stage III) breast cancers, we identified two somatic and one germline single nucleotide changes, each leading to amino acid substitution in the pRb protein (Leu607Ile, Arg698Trp, and Arg621Cys, respectively). This is the first study reporting point mutations affecting *RB1 *in breast cancer tissue. In addition, MLPA analysis revealed two large multiexon deletions (exons 13 to 27 and exons 21 to 23) with the exons 21-23 deletion occurring in the tumor also harboring the Leu607Ile mutation. Interestingly, Leu607Ile and Arg621Cys point mutations both localize to the spacer region of the pRb protein, a region previously shown to harbor somatic and germline mutations. Multiple sequence alignment across species indicates the spacer to be evolutionary conserved. All three *RB1 *point mutations encoded nuclear proteins with impaired ability to induce apoptosis compared to wild-type pRb *in vitro*. Notably, three out of four tumors harboring *RB1 *mutations displayed primary resistance to treatment with either 5-FU/mitomycin or doxorubicin while only 14 out of 64 tumors without mutations were resistant (p = 0.046).

**Conclusions:**

Although rare, our findings suggest *RB1 *mutations to be of pathological importance potentially affecting sensitivity to mitomycin/anthracycline treatment in breast cancer.

## Background

The retinoblastoma gene *(RB1) *is a tumor suppressor gene. pRb, the protein coded for by the *RB1 *gene, plays a pivotal role in cell cycle regulation, promoting G1/S arrest and growth restriction through inhibition of the E2F transcription factors [1]. Germline mutations affecting the *RB1 *gene are strongly associated with retinoblastoma development in children, and recent evidence has revealed an increased risk of different malignancies, including breast cancers, among patients cured from hereditary retinoblastoma [2].

Somatic alterations of the *RB1 *gene have been detected in different malignancies [3-5]. Previous studies have reported allelic imbalance (AI), loss of pRb protein expression [3], hypermethylation of the *RB1 *promoter [6] and, in some rare cases, large intragenic deletions [7] in the *RB1 *gene in primary breast cancer. However, point mutations (1163T>C and 1544C>T) have, so far, only been detected in a single breast cancer cell line (BT20) [8]. To the best of our knowledge, no point mutations have previously been reported in biopsies from breast carcinomas.

While the cellular functions of pRb are well characterized, the effect of disturbances in the *RB1 *gene on tumor growth and response to systemic therapy in breast cancer is incompletely understood. Lack of pRb protein and loss of heterozygosity (LOH) at the *RB1 *locus have been related to triple negative (TNBC) or basal cell-like breast cancer [9,10]. Absence of pRb expression has been linked to poor prognosis in breast cancer patients receiving adjuvant endocrine therapy [11,12]. In contrast, loss of expression has been associated with good prognosis in patients receiving chemotherapy [10,12]. However, these findings may not be interpreted as direct evidence that alterations in *RB1 *predict chemosensitivity [13]. Breast cancer patients are selected for systemic treatment options based on tumor characteristics like histological grading, estrogen receptor expression, and Her-2 status, thus, the patient cohorts referred to above may differ with respect to key biological parameters. Experimental studies have provided contradictory results, revealing loss of pRb function to enhance [11,14-17] as well as to reduce [18,19] cell death and sensitivity to chemotherapeutic agents.

In the current study, we analyzed 73 breast cancers undergoing pre-surgical treatment with doxorubicin or mitomycin with 5-FU for genetic and epigenetic changes in the *RB1 *gene. We report for the first time point mutations affecting *RB1 *in breast cancer tissue. Each mutation lead to amino acid substitution (Leu607Ile, Arg698Trp, and Arg621Cys) in pRb. The mutated pRb variants were all located to the nuclear compartment and expressed reduced apoptotic capacity compared to wild-type pRb. Furthermore, MLPA unveiled two large multiexon deletions (exons 13 to 27 and exons 21 to 23). Most interesting, three out of four tumors harboring *RB1 *mutations expressed resistance to chemotherapy. Our data provide the first indication that *RB1 *might be a candidate gene involved in drug resistance.

## Results

### Sequencing the *RB1 *coding exons

**Figure 1:**
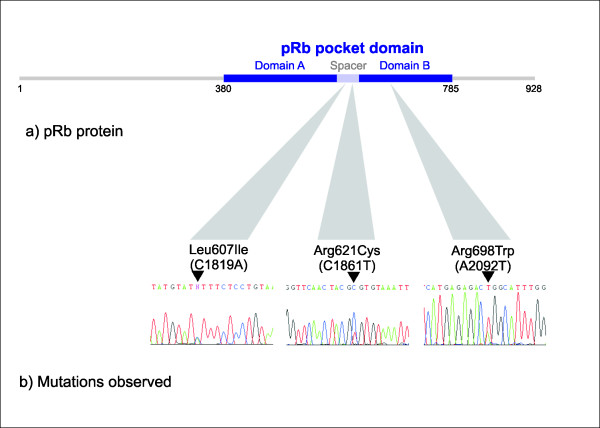
**Observed *RB1 *mutations**. (a) Schematic representation of the pRb protein. (b) We observed three novel point mutations in *RB1*: The C1819A mutation (Leu607Ile), the C1861T mutation (Arg621Cys), and the A2092T mutation (Arg698Trp), all leading to amino acid substitutions inside the pRb pocket domain.

cDNA generated from 73 locally advanced breast cancer samples obtained prior to chemotherapy was analyzed by PCR and DNA sequencing for *RB1 *mutations. Three tumors were found to harbor a single nucleotide change each, all resulting in amino acid substitutions (Table 1). Each mutation was located within the pocket domain of pRb (Figure 1). Two of the mutations were located in exon 19: C1819A (Leu607Ile) and C1861T (Arg621Cys), while the third was located in exon 20: A2092T (Arg698Trp) (nucleotide numbering according to GeneBank sequence L11910 with the A of ATG = number one). Each mutation was verified by amplification and sequencing of genomic DNA from the corresponding tumor.

**Table 1:** *RB1 *alterations observed among patients included in the study

Patient^1^	Alterations in *RB1*		Response^4^
		
	Large rearrangements^2^	Point Mutations^3^	
Dox19	AI	WT	PD
FUMI 12	AI	WT	PD
Dox 95	Del exon 13-27	WT	PD
FUMI 07	Del exon 21-23	Leu607Ile	PD
Dox 48	Duplication	WT	PD
Dox 65	WT	Arg621Cys (G.l)	PD
FUMI 39	Duplication	WT	SD
Dox 04	AI	WT	SD
Dox 32	AI	WT	SD
Dox 74	AI	WT	SD
Dox 83	AI	WT	SD
FUMI 25	AI	WT	SD
FUMI 26	AI	WT	SD
FUMI 27	AI	WT	SD
FUMI 44	AI	WT	SD
Dox 15	AI	WT	PR
Dox 109	AI	WT	PR
FUMI 15	AI	WT	PR
FUMI 23	AI	WT	PR
FUMI 29	AI	WT	PR
FUMI 37	AI	WT	PR
Dox 111	AI	Arg698Trp	PR
Dox 39	Duplication	WT	PR
FUMI 30	AI	WT	NE

^1 ^Dox "X", patients treated with doxorubicin; FUMI "X", patients treated with FUMI.

^2^AI, allelic imbalance; Del, deletion; WT, wild-type. ^3^G.l, germline. ^4 ^PD, progressive disease; SD, stable disease; PR, partial response; NE, not evaluable.

To evaluate whether the mutations were somatic or germline, DNA from corresponding lymphocytes were analyzed. One of these nucleotide changes, C1861T (Arg621Cys), was detected in white blood cells from the affected patient. This patient revealed no family history suggesting hereditary retinoblastoma. Thus, to evaluate whether the Arg621Cys alteration was a common polymorphism, we sequenced DNA from 231 healthy individuals. None of these samples harbored the C1861T nucleotide substitution, arguing against the hypothesis that C1861T could be a polymorphism occurring among >1% of the population (p < 0.10).

### Promoter analysis

**Figure 2:**
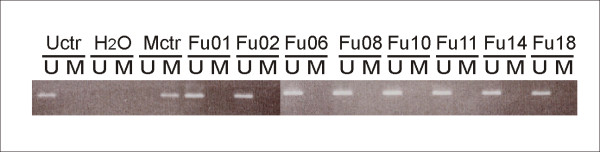
**Methylation of the *RB1 *promoter**. *RB1 *promoter methylation status was analyzed by methylation-specific PCR in 71 patients. Genomic DNA from patients was bisulfite converted. Figure shows products from MSP and USP of a representative selection of patients. Uctr: DNA from healthy donor; Mctr: Universally Methylated Control DNA.

In order to explore the occurrence of promoter aberrations as a possible cause for *RB1 *inactivation, 71 tumors were analyzed for *RB1 *promoter hypermethylation using methylation specific PCR, and 45 tumors were examined for mutations of the *RB1 *promoter by sequencing. No hypermethylation (Figure 2) or mutations of the *RB1 *promoter were detected.

### Analysing for large exon deletions with use of MLPA

Multiplex ligation-dependent probe amplification (MLPA) analysis was performed on tumor DNA from 71 of the samples including the tumors harboring point mutations. For the remaining two samples, genomic DNA was not available. Two individual samples harbored one large multiexon deletion each (exons 13 to 27 and exons 21 to 23), the exons 21-23 deletion occurring in the tumor also harboring the Leu607Ile point mutation (Table 1).

### Allelic imbalance

MLPA further revealed the *RB1 *gene to be duplicated in three of the samples (4%), and 18 of the tumors (25%) harbored a reduced copy number at the *RB1 *locus (Table 1).

In order to confirm the findings obtained by MLPA, traditional LOH analysis with VNTR/microsatellites was performed for all patients from whom white blood cell DNA was available (n = 43). Allelic imbalance at 13q14 was examined using three markers: D13S263 (located centromeric to *RB1*), D13S153 (located within intron 2 of *RB1*), and RB1 (located within intron 20 of *RB1*). For the informative samples, the findings detected by MLPA were confirmed in all cases (data not shown).

### *RB1 *mutations and response to chemotherapy *in vivo*

In this study, all breast cancer tissue samples analyzed (Additional file 1) were obtained from locally advanced primary breast cancers treated in two prospective translational phase III studies [20,21], aiming at identifying markers predicting therapy resistance [22]. All patients included from both protocols are listed in Additional file 1 together with their response to therapy. The total material of 73 tumors included 37 tumors from patients treated with 5-FU and mitomycin in concert (FUMI); eight out of these tumors expressed primary therapy resistance [21]. The remaining 36 patients were selected from a second study exploring resistance to weekly doxorubicin (Dox) among a total of 90 patients [20]. The sub-cohort analyzed here (n = 36) contained 9 tumors expressing primary therapy resistance towards doxorubicin together with a random set of 27 patients having an objective response to or stabilization of disease during doxorubicin treatment.

Notably, three out of four tumors harboring *RB1 *mutations (all tumors except the one harboring the Arg698Trp mutation) expressed primary resistance to therapy (Table 1 and Additional file 1). Thus, among 68 tumors analyzed by MLPA and cDNA sequencing for which clinical data on response was available, three out of a total of 17 tumors resistant to therapy (PD) harbored *RB1 *mutations, contrasting only one out of 51 tumors with stable disease or an objective response (p = 0.046). In contrast, no correlation between *RB1 *allelic imbalance and treatment response was found, and neither mutational status nor AI correlated to overall survival.

### Multiple sequence alignment of the pRb spacer

**Figure 3:**
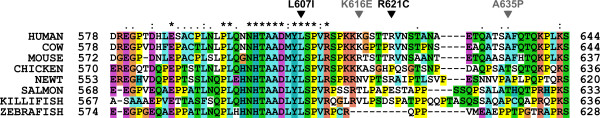
**Multiple sequence alignment of the pRb spacer region**. A multiple sequence alignment of the spacer region between the A and B boxes of the pRb pocket was generated with ClustalX using default parameters [24]. The locations of two of the mutations reported here are marked with black arrows, and two previously reported mutations are shown in grey [32,33]. Sequences from eight different species were included in the alignment: Human [UNIPROT: P06400 RB_HUMAN], cow [UNIPROT: Q08E68_BOVIN], mouse [UNIPROT: P13405 RB_MOUSE], chicken [UNIPROT: Q90600 RB_CHICK], newt [UNIPROT: Q98966_NOTVI], salmon [UNIPROT: C0H9R0_SALSA], killifish [UNIPROT: Q5J3Q9_FUNHE], and zebrafish [UNIPROT: A0JMQ4_DANRE].

The two point mutations Leu607Ile and Arg621Cys are both located in the spacer region, previously assumed to be non-essential to pRb protein function [23]. Employing ClustalX using default parameters [24], a multiple sequence alignment of the pRb spacer region including sequences from eight different species was constructed. As shown in Figure 3, the spacer region is fairly well conserved. In fact, the human and mouse *RB1*-spacer sequences have a higher level of identity than the average human-mouse sequence identity (82% versus 70%). This finding indicates that the spacer region is of important for pRb function.

### *In silico *structural modeling analysis

**Figure 4:**
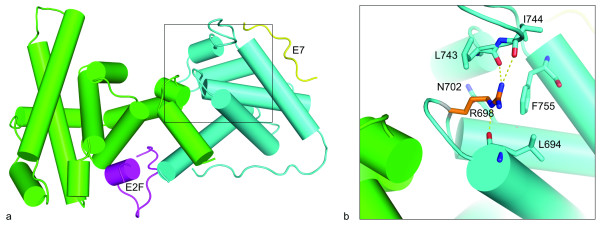
**Structural model of the pRb pocket domain**. (a) The cartoon shows the pRb pocket in complex with peptides from the transcription factor E2F (magenta) and the human papilloma virus protein E7 (yellow). The A and B boxes are colored green and cyan, respectively. The model was made from two structures: PDB: 1GUX (Rb pocket and E7 peptide) and PDB: 1O9K (E2F peptide). (b) Close-up view of the arginine 698 (R698) and amino acids that have at least one atom within a 4Å distance to the side chain atoms of R698. The R698 that is mutated to tryptophan in one of the tumors is located in the B box of the pRb pocket, and forms a hydrogen bond network with three backbone carbonyls. Hydrogen bonds to backbone carbonyls of residues L694, L743 and I744 are shown by yellow dotted lines. The structures were visualized using PyMOL http://www.pymol.org.

The Arg698Trp mutation is located in the B box of the pRb pocket (Figure 4a). *In silico *structural analysis of the pRb pocket [23] revealed the Arg698 residue to form a hydrogen-bond network (Figure 4b) and predicted Arg698Trp to disrupt this intramolecular hydrogen bond network with a possible structural and functional consequence on the pRb protein.

### Subcellular localization

**Figure 5:**
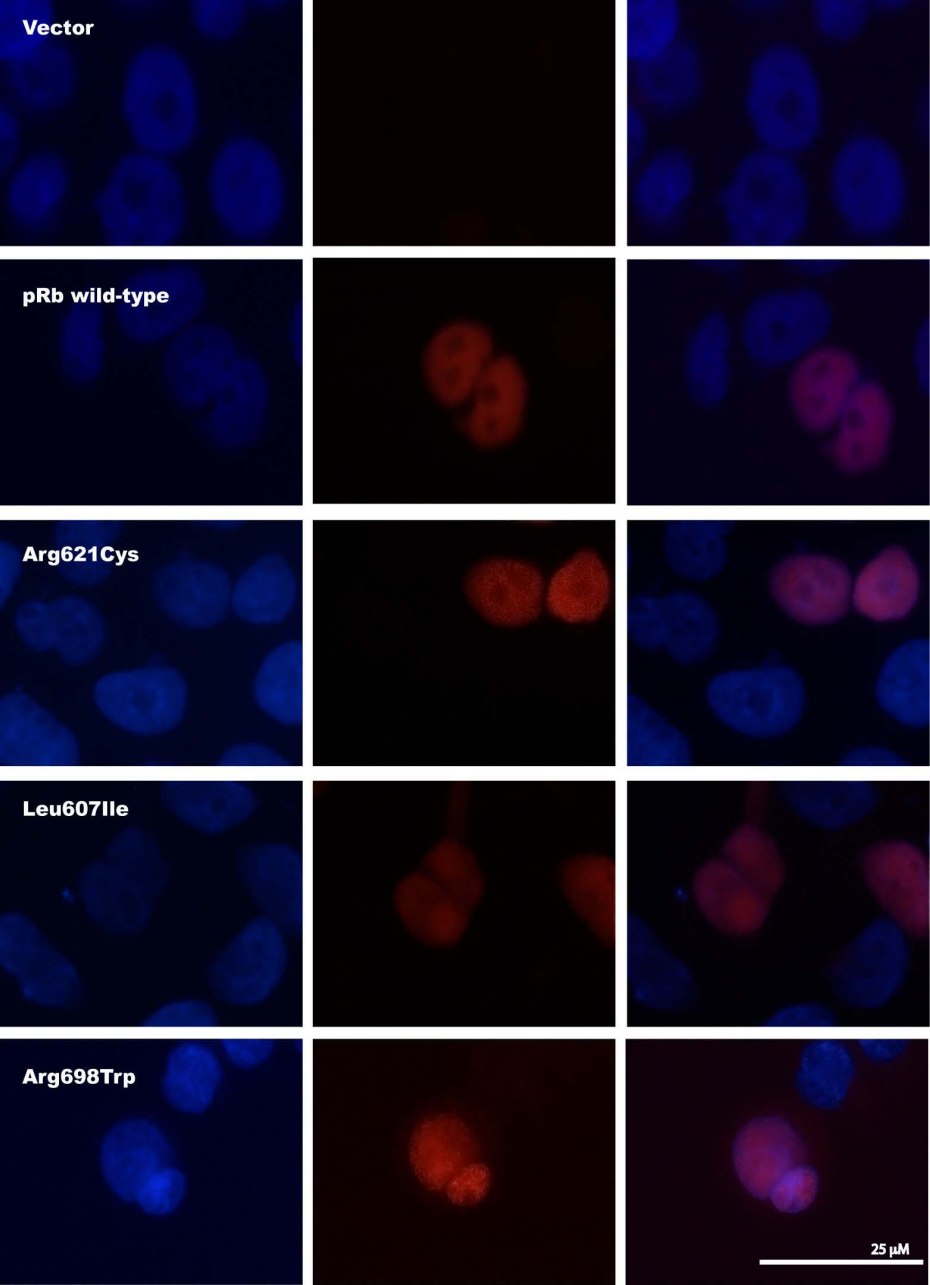
**Subcellular localization of pRb mutants**. Immunofluorescence staining of *RB1*-deficient C-33 A cells transfected with the pcDNA3.1/V5-His-TOPO vector or one of the four plasmids *RB1wild-type-V5*, *RB1Arg621Cys-V5, RB1Leu607Ile-V5, and RB1Arg698Trp-V5*. All mutants display nuclear localization. Left: DAPI, Centre: Anti-V5 (Texas Red), Right: Overlay.

Exploring expression of the mutant proteins in transfected *RB1*-deficient C-33 A cells, immunostaining revealed positive nuclear staining for all the three pRb point mutants (Leu607Ile, Arg698Trp, and Arg621Cys) similar as for pRb wild-type (Figure 5). Each control was negative with respect to unspecific fluorescence staining. This indicates that none of the mutants express altered activity due to improper subcellular localization.

### Apoptotic function and stability of the pRb mutant proteins

In addition to inhibit cell cycle progression, previous studies of the wild-type pRb protein have revealed pro-[19,25,26] as well as anti-[14-17] apoptotic functions in response to among others genotoxic stress like treatment with cytotoxic compounds *in vitro*.

**Figure 6:**
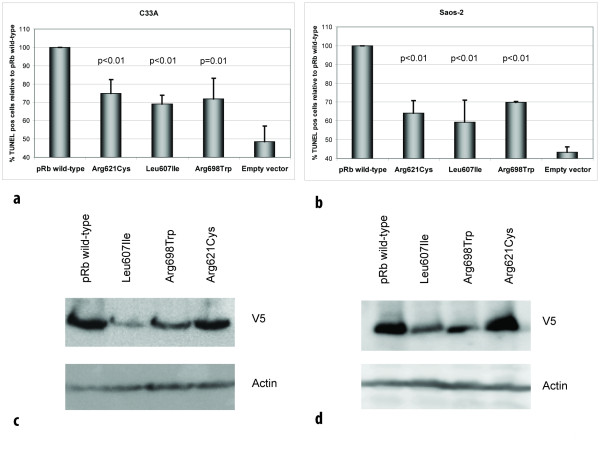
**Mutant pRb proteins display reduced apoptotic function**. (a) Diagram shows percentage of apoptotic (TUNEL positive) cells following doxorubicin treatment of *RB1-*deficient C-33 A and (b) Saos-2 cells, transfected with vectors expressing the different pRb mutants, relative to pRb wild-type (100%). For each mutant, a minimum of 1000 cells were counted in each of three independent experiments. Error bars indicate standard deviations between the three experiments. p-values given are based on analyses of variance (ANOVA) between the individual mutants and pRb wild-type. All over, each mutants revealed reduced apoptotic function in both cell lines (p ≤ 0.01). (c) Western blot controls for TUNEL assay. Panels displaying expression of pRb wild-type and mutants with corresponding actin expression (loading control) from transfections performed in parallel to those used for TUNEL assays in C-33 A and (d) Saos-2.

As two of the three point mutations were observed in patients not responding to DNA damaging chemotherapy, we aimed at exploring the ability of the wild-type and mutant pRb proteins to mediate apoptosis in *RB1*-deficient C-33 A cells following treatment with doxorubicin. Analysis by TUNEL assay revealed transfection of wild-type pRb to restore apoptosis in response to doxorubicin treatment in C-33 A cells (Figure 6a). While each of the point mutated pRb variants expressed some pro-apoptotic function, this was significantly reduced as compared to wild-type protein. Notably, these observations were confirmed in a second *RB1*-deficient cell line (Saos-2; Figure 6b) revealing the reduced pro-apoptotic effect to occur independent of cell line used (p ≤ 0.01 for all comparisons).

**Figure 7:**
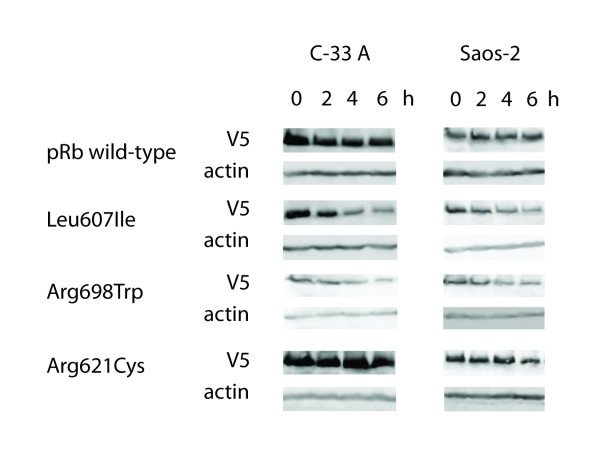
**Stability of pRb wild-type and mutant proteins**. Panels show the protein levels of pRb wild-type and mutants including corresponding actin levels as loading control. The samples were harvested 0-6 h after addition of cycloheximide (50 ug/ml) to transfected C-33 A and Saos-2 cells and examined by SDS-PAGE and western blot analyses.

Following plasmid transfection with identical amounts of DNA, western blot analyses on samples run in parallel with the TUNEL assays revealed the Leu607Ile and Arg698Trp mutants to reach lower protein amounts as compared to wild-type protein and the Arg621Cys mutant (Figure 6c and 6d). Protein measurement following cycloheximide treatment (Figure 7) revealed both Leu607Ile and Arg698Trp to display reduced stability, thus explaining the mechanism for reduced protein levels.

Consistent with the observed apoptotic responses, cell transformation experiments using NIH 3T3 cells revealed a slight, but not significant, increase in foci formation in cells transfected with plasmids carrying the mutant *RB1 *genes compared to wild-type *RB1 *(data not shown).

## Discussion

The present work is the first study reporting *RB1 *point mutations in primary breast carcinomas. Based on our findings that all three point mutated pRb proteins expressed reduced pro-apoptotic effect *in vitro*, and three out of four tumors harboring *RB1 *mutations were resistant to chemotherapy, our data provide the first indication that *RB1 *alterations could influence breast cancer chemosensitivity *in vivo*.

Somatic point mutations in *RB1 *have been detected in different malignancies including bladder [4] and prostate [5] cancer. While previous studies have examined breast cancer samples with respect to *RB1 *LOH, loss of pRb immunostaining [3] and chromosomal rearrangements [7], except for one study reporting no mutations when sequencing the exon 21 only [27] and the finding of point mutations in a single breast cancer cell line [8], we are not aware of any study reporting *RB1 *point mutations in breast cancer tissue http://rb1-lsdb.d-lohmann.de/.

In this study, we evaluated potential point mutations through gene sequencing (N = 73) and intragenetic deletions by use of MLPA (N = 71) across the whole coding region of the *RB1 *gene in stage III breast cancers. We identified three novel single nucleotide mutations in *RB1*, each leading to amino acid substitutions. While one of the point mutations was germline, the patient harboring this alteration revealed no family history of retinoblastoma or family clustering of either breast cancer or any other malignancy.

Each of the detected point mutations was located in the functionally important pocket domain. The pocket is essential for pRb's interaction with other proteins [28]. It consists of the so-called A and B boxes [23], forming a tight hydrophobic interface, with the two parts being covalently linked by the spacer region (Figure 1). Thus, mutations affecting the A [29] and B [30] boxes of pocket domain are known to give rise to pRb proteins with disturbed function. Alterations located in both of these parts have been detected in human sporadic cancers [4,5], but also as germline mutations in patients with retinoblastoma [31,32]. One of the point mutations identified here (Arg698Trp) is located in the B box, and *in silico *structural analysis suggested this mutation to disrupt a hydrogen-bond network. Furthermore, our findings indicate this mutant to be less stable than the wild-type pRb protein (Figure 7). Taken together, these findings suggest that mutations in this location might significantly affect pRb function and stability. In contrast, mutations Leu607Ile and Arg621Cys locate to the spacer region. While germline [31-33] as well as somatic [5] mutations within the spacer region have been identified, many authors have considered this domain non-essential to peptide-binding activity of the pRb pocket [23]. Our alignment analysis confirmed the spacer domain to be well conserved across different species (Figure 3), and the Leu607Ile mutant protein was considerably less stable compared to pRb wild-type These findings, in addition to our results revealing both of these mutations, similar to Arg698Trp, to express a reduced apoptotic function, further indicates a functional role for this region of the pRb pocket.

Considering the intragenic deletions (exon 21-23 and exon 13-27), these deletions result in truncated proteins missing half of the B box and both A and B boxes respectively, most likely abolishing the function of the pRb pocket in both cases.

The retinoblastoma gene encodes a nuclear phosphoprotein which in its unphosphorylated state binds to and inactivates E2F1, causing G1-S phase arrest [1]. Potential additional roles, including pro-apoptotic functions of pRb have been suggested [26]. While most studies have indicated an anti-apoptotic role of pRb [34], some studies have shown pRb to enhance apoptosis following γ-irradation [25] as well as doxorubicin-induced DNA damage [19] which is more in accordance with its role as a tumor suppressor. Here we found the apoptotic response to doxorubicin treatment to be restored by transfecting the *RB1*-deficient C-33 A and Saos-2 cell lines with wild-type pRb, but only to a minor degree when transfecting the pRb point mutants, supporting a pro-apoptotic function of pRb in response to anthracycline therapy (Figure 6a and 6b). However, the corresponding protein level controls and subsequent stability assays showed that the Leu607Ile and Arg698Trp pRb mutants were less stable than the mutant Arg621Cys pRb and the pRb wild-type proteins which harbored similar protein levels and stabilities. Thus, we believe the relatively low percentage of apoptotic cells observed for all the 3 mutants is due to reduced protein stability in the case of Leu607Ile and Arg698Trp, and due to impaired ability to induce apoptosis in the case of the more stable pRb mutant Arg621Cys.

Further, in support of a pro-apoptotic role of the pRb protein in breast cancer, three out of four tumors harboring *RB1 *mutations expressed resistance to doxorubicin or mitomycin treatment *in vivo*. The two drugs have similarities with respect to their mechanisms of antitumor actions. Thus, the *in vitro *findings on apoptotic response are consistent with our observations on patient drug resistance *in vivo*. The fact that one of the tumors (Dox 111 harboring Arg698Trp) responded to chemotherapy do not refute such a hypothesis; while we previously reported mutations affecting *TP53 *[20,21] to be associated with chemoresistance *in vivo*, the predictive power was not 100%; indicating alternative mechanisms may act in concert [35].

## Conclusions

In summary, we demonstrate for the first time point mutations in *RB1 *among breast cancer tumors. Most interestingly, these point mutated genes encode proteins expressing reduced pro-apoptotic effect *in vitro*, and three out of four tumors harboring *RB1 *mutations were resistant to chemotherapy. Chemoresistance remains the main obstacle to cure breast cancer as well as most other solid malignancies. Although *RB1 *mutations were detected in a minority of tumors revealing chemoresistance *in vivo *(3/17), our present findings with respect to specific *RB1 *mutations affecting apoptotic response to doxorubicin treatment may point to functional network(s) potentially important for drug resistance *in vivo*. These findings merit further investigations of factors involved in the *RB1 *pathway and their role in chemotherapeutic response in breast cancer.

## Methods

### Patients

This study included patients from two prospective studies addressing the potential role of mutations in *TP53 *and other genes regarding resistance to treatment with doxorubicin [20] or mitomycin and 5-fluorouracil [21] in locally advanced breast cancer. Both studies were approved by the Regional Ethical Committee, and each patient gave written informed consent to the procedure. Because these studies were designed to explore causes of chemoresistance, we focused on comparing tumors that showed primary drug resistance (progressive disease within 12 weeks) with the combined group of tumors showing stable disease or objective response [22,36]. Thus, for the patients treated with doxorubicin, we analyzed *RB1 *mutational status in all nine tumors that were resistant to therapy together with a randomly selected subgroup of 27 responding tumors. Regarding the group of patients treated with mitomycin and 5-fluorouracil, we analyzed 37 tumors for the presence of *RB1 *mutations, including eight patients resistant to therapy [21] and four none evaluable samples. Included were three patients with locally advanced breast cancer, treated with mitomycin and 5-FU, not participating in these studies. Thus, a total of 73 patients were included (Additional file 1).

### Control subjects

Due to the fact that we discovered a novel germline base substitution C1861T (Arg621Cys) in one breast cancer patient, we sought to evaluate its frequency in the general Norwegian population. Thus, we examined blood DNA from 231 healthy women recruited from the national mammographic program into other studies described elsewhere [37].

### RNA Purification

RNA was purified by Trizol (life Technologies, Inc.) extraction from snap-frozen tissue samples according to the manufacturer's instructions. After extraction, the RNA was dissolved in diethyl pyrocarbonate-treated double-distilled, deionized H _2_O. cDNA was synthesized by reverse transcription using Superscript II reverse transcriptase (Invitrogen)

### DNA Purification

Genomic DNA was purified from tumor tissue or lymphocytes using QIAamp DNA Mini Kit (Qiagen) according to the manufacturer's instructions.

### Multiplex ligand probe amplification (MLPA)

MLPA analysis of genomic DNA from 71 patients was performed using the SALSA MLPA *RB1 *kit (MRC-Holland, Amsterdam, The Netherlands) according to the manufacturer's instructions. In the patient samples, the peak areas of all MLPA products resulting from *RB1 *specific probes were first normalized by the average of peak areas resulting from control probes specific for locations other than on chromosomes 13. A ratio was then calculated where this normalized value was divided by the corresponding value from a sample consisting of pooled DNA from 10 healthy individuals. A sample was scored as having a reduced copy number at a specific location if this ratio was below 0.75, and as having an increased copy number if the ratio was above 1.25.

### LOH analysis

Samples shown by MLPA analysis to harbor AI at the *RB1 *locus were subsequently analyzed by LOH. Three markers were applied spanning 13q14.1-3: D13S263, centromeric to *RB1*, D13S153, and RB1, the two latter located within intron 2 and intron 20 of the *RB1 *gene, respectively. Both D13S263 and D13S153 are microsattelite markers; while RB1 is a variable number tandem repeat (VNTR). All three markers were amplified by PCR using primers as specified in Table 2 and the PCR products were analyzed on an automated DNA sequencer (ABI 3700). Data were analyzed by comparing normal and tumor tissue allele peak-height ratios. A sample was scored as having AI when the ratio between height of tumor and normal sample was less than 0.84 [38].

**Table 2:** PCR and DNA sequencing primers

Primer name	Sense primer	Anti sense primer	Length (bp)	Annealing Temp. (°C)
RB1M	5'-GGG AGT TTC GCG GACGTG AC-3'	5'-ACG TCGAAA CAC GCC CCG-3'	172	65.0
RB1U	5'-GGG AGT TTT GTG GAT GTG AT-3'	5'-ACA TCAAAA CAC ACC CCA-3'	172	61.0
RB1 promoter	5'-CGC CCC AGT TCC CCA CAG A-3'	5'-GGC AAC TGA GCG CCG CGT-3'	163	53.0
RB1 exon 1	5'-AAC GGG AGT CGG GAG AG-3'	5'-AAT CCT GTC ACC ATT CTG C-3'	412	55.2
RB1 exon 2	5'-GAT TAT TTT CAT TTG GTA GGC-3'	5'-AAA GTG GTA GGA TTA CAG GC-3'	351	51.3
RB1 exon 3	5'-TTT TAA CAT AGT ATC CAG TGT GTG-3'	5'-TAC ACT TTC ATA ACG GCT CC-3'	350	54.4
RB1 exon 4	5'-GAC CCT TCG TTT TCT TAT ATT CTC-3'	5'-ATC AGA GTG TAA CCC TAA TAA AAT G-3'	390	55.2
RB1 exon 5	5'-ATT GGG AAA ATC TAC TTG AAC-3'	5'-TCA AAC TAA CCC TAA CTA TCA AG-3'	265	54.2
RB1 exon 6	5'-CAT TCT ATT ATG CAT TTA ACT AAG G-3'	5'-CTA ACA GTT AAT AAG CCA AGC AG-3'	340	53.6
RB1 exon 7	5'-ATG GAT ATA CTC TAC CCT GCG-3'	5'-ATC CTG TCA GCC TTA GAA CC-3'	291	55.2
RB1 exon 8	5'-TAA AAG TAG TAG AAT GTT ACC AAG-3'	5'-CAG TGA TTC CAG AGT GAG G-3'	470	55.2
RB1 exon 9	5'-TTG ACA CCT CTA ACT TAC CCT G-3'	5'-TTG GCT AGA TTC TTC TTG GG-3'	301	55.7
RB1 exon 10	5'-GAA ATC TGT GCC TCT GTG TG-3'	5'-AAA GGT AAC TGT TAT AGG ACA CAC-3'	200	52.9
RB1 exon 11	5'-GTT ATC AAT ACC ACC AGG GAG-3'	5'-CAA ATC TGA AAC ACT ATA AAG CC-3'	443	53.0
RB1 exon 19	5'-AGA CAA GAT GTA TCT GGG TGT AC-3'	5'-CAT GAT TTG AAC CCA GTC AG-3'	306	53.6
RB1 exon 20	5'-CTT ATT CCC ACA GTG TAT GCC-3'	5'-AGC CTG GGT AAC AGA GTG AG-3'	341	47.5
RB1 exon 21	5'-ATT CTG ACT ACT TTT ACA TC-3'	5'-TTA TGT TAT GGA TAT GGA T-3'	192	58.0
RB1 exon 22.1	5'-ATA TGT GCT TCT TAC CAG T-3'	5'-CAC GTT TGA ATG TCT GAG GA-3'	148	53.5
RB1 exon 22.2	5'-CCT CAG ACA TTC AAA CGT GT-3'	5'-TTG GTG GAC CCA TTA CAT TA-3'	175	54.1
RB1 exon 23	5'-TAA TGT AAT GGG TCC ACC AA-3'	5'-TCA AAA TAA TCC CCC TCT CA-3'	277	55.6
RB1 exon 24	5'-GAA TGA TGT ATT TAT GCT CA-3'	5'-TTC TTT TAT ACT TAC AAT GC-3'	165	46.1
RB1 exon 25	5'-CTT TGC CTG ATT TTT GAC AC-3'	5'-CAG TGC TGA GAC TCT GGA TTC-3'	270	56.3
RB1 exon 26	5'-CAT TTA TGT TTT AGA TGG TTA G-3'	5'-GTT TAT TTC GTT TAC ACA AG-3'	318	46.8
RB1 exon 27	5'-CAG CCA CTT GCC AAC TTA C-3'	5'-CAT AAA CAG AAC CTG GGA AAG-3'	230	53.5
RB1-1.r-S2/AS4	5'-AAC GGG AGT CGG GAG AG-3'	5'-GAA TTA CAT TCA CCT CTT CAT CAA G-3'	1204	45.0
RB1-1.r-S3/AS2	5'-ATG ATA AAA CTC TTC AGA CTG ATT C-3'	5'-TGT CCA CCA AGG TCC TGA G-3'	1794	45.0
RB1-2.r-frag1-S/AS	5'-AGG AGG ACC CAG AGC AGG AC-3'	5'-CCA AGA AAC TTT TAG CAC CAA TG-3'	496	45.0
RB1-2.r-frag2-S/AS	5'-CTA CTG AAA TAA ATT CTG CAT TGG T-3'	5'-CTC TTC ATC AAG GTT ACT TTT TCG T-3'	528	45.0
RB1-2.r-frag3-S/AS	5'-GAA ACA CAG AGA ACA CCA C-3'	5'-ATT CTG AGA TGT ACT TCT GCT A-3'	461	45.0
RB1-2.r-frag4-S/AS	5'-AGC AAA CTT CTG AAT GAC AAC-3'	5'-GAG AGG TAG ATT TCA ATG G-3'	518	45.0
RB1-2.r-frag5-S/AS	5'-CTC CAA AGA AAA AAG GTT CAA-3'	5'-GGT ATT GGT GAC AAG GTA GG-3'	512	45.0
RB1-2.r-frag6-S/AS	5'-GTA TTC TAT AAC TCG GTC TTC A-3'	5'-CAT TTC TCT TCC TTG TTT GA-3'	526	45.0
RB1-plasmid-S1/AS1	5'-GGT TTT TCT CAG GGG ACG-3'	5'-GTG AGA GAC AAT GAA TCC AGA G-3'		45.0
RB1-plasmid-S/AS-STOP	5'-CAC AGC TCG CTG GCT CCC-3'	5'-TTT CTC TTC CTT GTT TGA G-3'		45.0
RB1 (Leu607Ile)	5'-GCA GCA GAT ATG TAT ATT TCT CCT GTA AGA TCT CC-3'			
RB1 (Arg621Cys)	5'-AAA GGT TCA ACT ACG TGT GTA AAT TCT ACT GC-3'			
RB1 (Arg698Trp)	5'-GAA CTC ATG AGA GAC TGG CAT TTG GAC CAA ATT ATG-3'			
D13S153 F/R	5'-TTG CAC TGT GGA GAT AAA CAC ATA G-3'	5'-TCA CAT TGT CTT TTA AGG CAG GAG-3'		
D13S263 F/R	5'-CCT GGC CTG TTA GTT TTT ATT GTT A-3'	5'-CCC AGT CTT GGG TAT GTT TTT A-3'		
RB1	5'-TGT ATC GGC TAG CCT ATC TC-3'	5'-AAT TAA CAA GGT GTG GTG G-3'		

### PCR Amplification of *RB1 *from cDNA

Six fragments encompassing the *RB1 *reading frame starting from nucleotide 89 to 2749 (TGA) were amplified by nested PCR using the primers listed in Table 2. The nucleotides 1 (starting from ATG) to 88 were not covered by our analysis. The frequency of published mutations in this region is approximately 2% for patients with retinoblastoma http://rb1-lsdb.d-lohmann.de/. It was therefore concluded that the risk of missing somatic alterations was acceptably low. Observed mutations were verified by PCR amplification of the affected exons from genomic DNA. In some tumors, no cDNA-based products were detected for parts of the *RB1 *gene. To verify the integrity of the *RB1 *gene in these patients, we amplified the exons of interest using genomic DNA.

PCR was carried out with Dynazyme EXT DNA polymerase (Dynazyme) in a 50 μl solution containing 1× PCR buffer, 1.5 mM MgCl _2_, 0.5 mM of each deoxynucleotide tri-phosphate, 5% DMSO, 0.2 μM of each primer, and 0.5 μl of cDNA, 1 μl of first-round PCR-product or ~50 ng genomic DNA was used as template. The *RB1 *PCR conditions for the first round of the nested PCR for fragments one and two were: An initial 5 min denaturation at 94°C, 40 cycles of 30 sec at 94°C, 30 sec at 52.5°C, and 120 sec at 72°C, and a final 7 min extension at 72°C. The second round of the nested PCR for fragments one and two was conducted as follows: An initial 5 min denaturation at 94°C, 40 cycles of 30 sec at 94°C, 30 sec at 55.6°C, and 60 sec at 72°C, and a final 10 min extension at 72°C.

The *RB1 *PCR conditions for both rounds of the nested PCR for fragments three to six were identical and consisted of: An initial 5 min denaturation at 94°C, 40 cycles of 30 sec at 94°C, 30 sec at 45°C, and 120 sec at 72°C, and a final 7 min extension at 72°C.

PCR on genomic DNA was done with an initial 5 min denaturation at 94°C, 40 cycles of 60 sec at 94°C, 30 sec at an annealing temperature optimized for each exon (Table 2), and 45 sec at 72°C, and a final 7 min extension at 72°C.

Finally, the *RB1 *promoter PCR was done with an initial 5 min denaturation at 94°C, 35 cycles of 30 sec at 94°C, 30 sec at 53°C, and 45 sec at 72°C, and a final 5 min extension at 72°C.

### DNA Sequencing

Sequencing was performed as described previously [39] using primers specific for the different *RB1 *fragments (Table 2). The sequences generated were compared with wild-type *RB1 *(GenBank accession number: L11910) for sequence alterations.

### Promoter Methylation Analysis

*RB1 *promoter methylation status was analyzed by methylation-specific PCR in 71 patients. Genomic DNA from patients was modified using the CpGenome DNA Modification Kit (Intergen), and primers designed specific for methylated (RB1M-S and RB1M-AS, Table 2) and unmethylated (RB1U-S and RB1U-AS, Table 2) DNA [40]. Methylation- and unmethylation-specific PCRs (MSP and USP) were done with AmpliTaq Gold DNA Polymerase (Applied Biosystems) in a 50 μl solution containing 1× PCR buffer, 1.5 mM MgCl _2_, 0.5 mM of each deoxynucleotide triphosphate, 0.2 μM of each primer and 25 ng of modified genomic DNA. The methylation-specific PCR was carried out for 35 cycles of 60 sec at 95°C, 45 sec at 65°C, and 1 min at 72°C. The unmethylation-specific PCR was carried out for 35 cycles of 60 sec at 95°C, 45 sec at 61°C, and 1 min at 72°C. Both PCRs were performed with an initial 5 min of denaturation at 95°C and concluded with 2 min at 72°C. After amplification, the PCR products were visualized on a 3% agarose gel. Included in each run was a positive control (CpGenome Universal Methylated DNA, Millipore) and two negative controls (modified DNA from healthy donors and water).

### Plasmid constructs

*RB1 *wild-type was amplified from cDNA by nested PCR using the primers RB1-plasmid-S1 and RB1-plasmid-AS1 in the first, and RB1-plasmid-S and RB-AS-STOP in the second PCR (Table 2). The first PCR was carried out in a 50 μl reaction mixture of 2.5 U KOD XL DNA polymerase (Novagen), 1× PCR buffer, 0.2 mM of each deoxynucleotide triphosphate, 0.2 μM of each primer and 10 μl of cDNA. The thermal conditions for the first PCR were as follows: 94°C for 5 min, 30 cycles of 30 sec at 94°C, 2 sec at 45°C and 180 sec at 70°C and a final extension at 74°C for 10 min. For the second PCR, a 50 μl reaction solution was made consisting of 0.5 U Dynazyme EXT DNA polymerase, 1× PCR buffer, 5% DMSO, 0.2 mM of each deoxynucleotide triphosphate, 0.2 μM of each primer and 1 μl of first-round PCR product. The amplification conditions were: 5 min denaturation at 94°C followed by 40 cycles of 30 sec at 94°C, 30 sec at 45°C and 180 sec at 72°C before a final step at 72°C for 7 min.

The final PCR product was TA-cloned into the expression vector pcDNA3.1/V5-His TOPO (Invitrogen) according to the manufacturer's instructions. Using the resulting construct *RB1wild-type-V5*, corresponding primers (Table 2), and QuickChange Multi Site-Directed Mutagenesis Kit (Stratagene), the following constructs were made; *RB1Arg621Cys-V5, RB1Leu607Ile-V5, and RB1Arg698Trp-V5*.

### Cell culture and transfection

C-33 A cells and Saos-2 cells were cultured in EMEM and McCoys 5A, respectively, supplemented with 10% FBS. Both cell lines were purchased from ATCC (American Type Culture Collection). Transfection was performed using Lipofectamine 2000 (Invitrogen) according to the manufacturer's instruction.

### Western blot analysis

Protein samples were separated on 8% SDS-polyacrylamide gels and blotted onto nitrocellulose membranes (Whatman). The membranes were probed with the following antibodies: anti-V5 (Invitrogen) and anti-actin (Santa Cruz Biotechnology), and the HRP-conjugated 2. antibodies sheep anti-mouse (GE Healthcare) and mouse anti-goat (Santa Cruz Biotechnology). Signals were detected using ECL Western blotting Detection Reagents (GE Healthcare).

### Protein stability

Analyses of protein stability using cycloheximide was performed as previously described [41]. After 0, 2, 4, and 6 h, the cells were harvested and lysed in 1 X SDS lysis buffer (0.075 M Tris pH 6.8, 2% SDS, 20% glycerol, 0.1 M β-mercaptoethanol, 0.01% bromphenol blue). The samples were analyzed by SDS-PAGE and western blot using anti-V5 and anti-actin (loading control).

### Immunofluorescence

Cells grown on cover slips were fixed in 3.7% formaldehyde for 20 minutes and washed with 1× PBS. The cells were permeabilized for 12 minutes using 0.1% triton X100 in 1× PBS before blocking with 1% BSA in 1× PBS. Detection of the corresponding proteins was performed using monoclonal mouse anti-V5 and TXR-conjugated goat anti-mouse Ig secondary antibody. The slides were mounted with Vectashield (Vector Laboratories, USA) and examined using a confocal laser scanning microscope (Leica TCS Confocal System attached to a Leica DM RXA microscope).

### Statistical Analysis

Alterations in the *RB1 *gene were correlated to response to chemotherapy by use of Fisher exact test (p-value given as cumulative, two-sided). Differences between pRb wild-type and mutants with respect to induction of apoptosis were evaluated by analyses of variance (ANOVA). Subject to statistical significance, the efficacy of each mutant was compared to pRb wild-type with use of the Student test for 2 samples. Statistical calculations were performed using the SPSS 15.0 software and http://www.quantitativeskills.com/sisa/.

### TUNEL assay

*RB1*-deficient C-33 A cells [26] were transfected with pcDNA3.1/V5-His-TOPO vector or one of the four constructs *RB1wild-type-V5*, *RB1Arg621Cys-V5, RB1Leu607Ile-V5, or RB1Arg698Trp-V5*. After 24 hours, the cells were treated with 5 μM doxorubicin (adriamycin) for 6 hours, and washed with 1× PBS before cytospins were prepared. The cells were fixed with 3.7% paraformaldehyde o.n at room temperature and permabilized with 0.1% Triton X-100 in sodium citrate for 2 min on ice. TUNEL reaction mixture (In situ Cell Death Detection Kit, TMR red, Roche) was applied for 2.5 h in dark at 37°C. Controls were either treated with DNaseI (positive control) or with a TUNEL reaction mixture not containing enzyme. Hoechst was used to visualize the nucleus and the cells were analyzed using Leica DMI6000B epifluorescence microscope (Leica Microsystems). A minimum of 1000 cells for all samples were counted in each of three independent experiments. The same experiment was repeated in Saos-2 cells. Here, the optimal conditions for detection of changes in pro-apoptotic function differed from C-33 A cells. Saos-2 cells were treated with 0.5 μM doxorubicin (adriamycin) for 12 hours before harvest.

## Abbreviations

(AI): Allelic imbalance; (Dox): Doxorubicin; (FUMI): 5-FU/Mitomycin; (LOH): Loss of heterozygosity; (MLPA): Multiplex ligation-dependent probe amplification; (MSP): Methylation-specific PCR; (pRb): Retinoblastoma gene product; (*RB1*): Retinoblastoma tumor suppressor gene; (USP): Unmethylation-specific PCR.

## Competing interests

The authors declare that they have no competing interests.

## Authors' contributions

EOB prepared the manuscript and was responsible for the immunofluoresence experiment. EOB and SK carried out the LOH, MLPA and TUNEL assays and the statistical calculations. SG performed the nucleic acids extraction, sequencing, and methylation experiments supported by VS and EOB. PP was responsible for the multiple sequence alignment and the *in silico *structural modeling analysis. MP contributed to the design of plasmid constructs, and ALBD was involved in the LOH analysis and helped drafting the manuscript. JRL supervised the laboratory analyses and participated in writing the paper. PEL directed the clinical study and provided the tumor samples. In addition PEL conceived and directed the project, and contributed to the writing of the manuscript. All authors have read and approved the manuscript.

## Supplementary Material

Additional file 1 **Characteristics of all patients included in the study**. Treatment, age, grade, stage of disease, clinical response and outcome of patients included in the study. Click here for file
