# Supplementary material for: Identification and characterization of retinoblastoma gene mutations disturbing apoptosis in human breast cancers
Source: Mol Cancer. 2010 Jul 1;9:173. doi: 10.1186/1476-4598-9-173 (PMC2908580; doi:10.1186/1476-4598-9-173)
Supplement: Additional file 1 — Characteristics of all patients included in the study. Treatment, age, grade, stage of disease, clinical response and outcome of patients included in the study. [file 1476-4598-9-173-S1.DOC]

**Additional file 1: Characteristics of all patients included in the study.**

| **Patient^1^** | **Age** | **Grade** | **Stage** | **Clinical** | **Outcome ^4^** |
| --- | --- | --- | --- | --- | --- |
|  |  |  | **of** | **response ^3^** |  |
|  |  |  | **disease ^2^** |  |  |
|  |  |  |  |  |  |
| Dox 011 | 67 | 2 | III | PD | BCD15 |
| Dox 019 | 74 | 3 | III | PD | BCD20 |
| Dox 026 | 72 | 2 | III | PD | R9 |
| Dox 048 | 54 | 3 | III | PD | R4 |
| Dox 057 | 46 | 3 | III | PD | BCD10 |
| Dox 065^5^ | 66 | 3 | III | PD | BCD23 |
| Dox 095^5^ | 75 | 3 | III | PD | BCD33 |
| Dox 104 | 44 | 3 | III | PD | F31 |
| Dox 112 | 37 | 1 | III | PD | F22 |
| FUMI 05 | 51 | 3 | III | PD | R40 |
| FUMI 07^5^ | 51 | 2 | III | PD | F46 |
| FUMI 12 | 73 | 2 | III | PD | BCD5 |
| FUMI 18 | 38 | 3 | IV | PD | BCD3 |
| FUMI 19 | 70 | 3 | IV | PD | BCD8 |
| FUMI 22 | 82 | 2 | III | PD | Non-BCD28 |
| FUMI 31 | 79 | 2 | III | PD | BCD12 |
| FUMI 45 | 78 | 2 | III | PD | F13 |
| Dox 004 | 68 | 2 | III | SD | F92 |
| Dox 005 | 61 | 3 | III | SD | R9 |
| Dox 021 | 79 | 3 | III | SD | R12 |
| Dox 032 | 55 | 1 | III | SD | F73 |
| Dox 037 | 71 | 3 | III | SD | R12 |
| Dox 051 | 53 | 3 | III | SD | F60 |
| Dox 055 | 71 | 2 | III | SD | R10 |
| Dox 063 | 44 | 3 | III | SD | R25 |
| Dox 074 | 79 | 1 | III | SD | Non-BCD40 |
| Dox 080 | 57 | 2 | III | SD | R9 |
| Dox 083 | 79 | 2 | IV | SD | M |
| Dox 090 | 64 | 2 | III | SD | F36 |
| Dox 092 | 63 | 3 | IV | SD | BCD29 |
| FUMI 04 | 37 | 3 | III | SD | R13 |
| FUMI 08 | 59 | 3 | III | SD | BCD10 |
| FUMI 09 | 63 | 2 | III | SD | F38 |
| FUMI 11 | 59 | 2 | III | SD | R38 |
| FUMI 14 | 70 | 2 | III | SD | F40 |
| FUMI 16 | 74 | 2 | IV | SD | M |
| FUMI 17 | 70 | 2 | III | SD | Non-BCD4 |
| FUMI 24 | 73 | 2 | IV | SD | M |
| FUMI 25 | 54 | 2 | IV | SD | M |
| FUMI 26 | 73 | 3 | IV | SD | BCD8 |
| FUMI 27 | 56 | 3 | III | SD | R7 |
| FUMI 39 | 74 | 3 | III | SD | F14 |
| FUMI 40 | 80 | 2 | IV | SD | M |
| FUMI 44 | 76 | 3 | III | SD | R14 |
| Dox 007 | 66 | 2 | III | PR | R39 |
| Dox 014 | 82 | 2 | III | PR | R11 |
| Dox 015 | 47 | 3 | III | PR | F79 |
| Dox 016 | 73 | 2 | III | PR | F78 |
| Dox 018 | 66 | 2 | III | PR | Non-BCD33 |
| Dox 027 | 78 | 1 | III | PR | R59 |
| Dox 039 | 85 | 2 | III | PR | F67 |
| Dox 053 | 32 | 2 | III | PR | R23 |
| Dox 061 | 58 | 3 | III | PR | F57 |
| Dox 075 | 56 | 1 | IV | PR | Non-BCD40 |
| Dox 100 | 47 | 3 | III | PR | R10 |
| Dox 101 | 73 | 2 | III | PR | R20 |
| Dox 109 | 48 | 3 | III | PR | F26 |
| Dox 111^5^ | 54 | 3 | III | PR | F30 |
| FUMI 01 | 58 | 3 | IV | PR | F54 |
| FUMI 02 | 44 | 3 | III | PR | F53 |
| FUMI 06 | 42 | 3 | III | PR | R13 |
| FUMI 15 | 70 | 2 | III | PR | R32 |
| FUMI 20 | 50 | 3 | III | PR | R14 |
| FUMI 23 | 65 | 3 | III | PR | F41 |
| FUMI 29 | 59 | 3 | III | PR | F26 |
| FUMI 37 | 67 | 2 | III | PR | F16 |
| FUMI 41 | 53 | 3 | IV | PR | M |
| FUMI 43 | 69 | 3 | III | PR | F15 |
| FUMI 49 | 59 | 3 | III | PR | F9 |
| FUMI 10 | 76 | 2 | III | NE | R37 |
| FUMI 21 | 74 | 3 | IV | NE | BCD9 |
| FUMI 30 | 40 | 3 | III | NE | BCD12 |
| FUMI 35 | 46 | 2 | III | NE | F17 |

*^1^* Dox “X”, patients treated with doxorubicin; FUMI ”X”, patients treated with FUMI. *^2^* primary stage III (T_3_/T_4_ or N_2_), primary stage IV (limited distant metastasis) at diagnosis. *^3^* PD, progressive disease; SD, stable disease; PR, partial response. *^4^* BCD”X”, breast cancer death after ”X” months; Non-BCD, patients died of other causes than breast cancer; F”X”, disease free after ”X” months of follow-up; R”X”, alive, but suffering a relapse after ”X” months; M, metastasis. *^5^*Patients harboring *RB1* mutations are colored blue.
